# Explainable Deep Learning for Research on the Synergistic Mechanisms of Multiple Pollutants: A Critical Review

**DOI:** 10.3390/toxics14040335

**Published:** 2026-04-16

**Authors:** Chang Liu, Anfei He, Jie Gu, Mulan Ji, Jie Hu, Shufeng Qiao, Fenghe Wang, Jing Hua, Jian Wang

**Affiliations:** 1 School of Chemistry and Chemical Engineering, Nanjing University of Science and Technology, Nanjing 210094, China; liuchang@nies.org (C.L.); wangfenghe@njust.edu.cn (F.W.); 2 Nanjing Institute of Environmental Sciences, Ministry of Ecology and Environment of China, Nanjing 210042, China; gujie@nies.org (J.G.); jimulan@nies.org (M.J.); hujie@nies.org (J.H.); qiaoshufeng@nies.org (S.Q.); 3 School of Environmental Science and Engineering, Suzhou University of Science and Technology, Suzhou 215009, China; heanfei@usts.edu.cn

**Keywords:** explainable deep learning, multiple pollutants, source apportionment, control strategy, model interpretation

## Abstract

The synergistic control of multiple pollutants is critically challenged by complex nonlinear interactions, strong spatiotemporal heterogeneity, and the difficulty of tracing causal drivers. Deep learning offers high predictive power but suffers from the “black-box” problem, limiting its acceptance in environmental decision-making. Explainable Deep Learning (XDL) integrates physical mechanisms with interpretable algorithms, achieving both prediction accuracy and explanatory transparency. This review systematically evaluates the effectiveness and limitations of XDL in analyzing multi-pollutant interactions, with a comparative focus on atmospheric and aquatic environments. Key techniques, including SHAP, attention mechanisms, and physics-informed neural networks, are examined for their roles in synergistic monitoring, source apportionment, and regulatory optimization. The main findings reveal that: (1) XDL, particularly the “tree model + SHAP” paradigm, has become a dominant tool for quantifying driving factors, yet most attributions remain correlational rather than causal; (2) physics-informed fusion (soft vs. hard constraints) improves physical consistency but faces unresolved conflicts between data and physical laws, with current models lacking a conflict detection mechanism; (3) cross-media comparison shows a unified technical logic of “physical mechanism guidance + post hoc feature attribution”, but atmospheric applications lead in embedding advection–diffusion constraints, while aquatic research excels in spatial topology modeling via graph neural networks; (4) critical bottlenecks include the lack of causal inference, uncertainty-unaware interpretations, and data scarcity. Future directions demand a shift from correlation-only to causal-aware attribution, from blind fusion to conflict-detecting systems, and from no evaluation standards to domain-specific validation benchmarks. XDL is poised to transform multi-pollutant governance from experience-driven to intelligence-driven approaches, provided that verifiable interpretability and physical consistency become core design principles.

## 1. Introduction

With accelerated industrialization and urbanization, environmental challenges have evolved from single-pollutant contamination to complex, multi-medium, multi-component, and multi-scale pollution [1]. Issues such as synergistic pollution of PM_2.5_ and ozone (O_3_) in the atmosphere, coexistence of heavy metals and persistent organic pollutants in water bodies, and cross-medium migration of pollutants in soil–groundwater systems have become increasingly prominent, posing severe threats to ecological security and public health [2,3,4].

Traditional multi-pollutant management relies on physicochemical models and linear statistical methods, which struggle to accurately capture nonlinear interactions between pollutants and migration/transformation patterns under complex environmental conditions. Deep learning technology enables deep mining of high-dimensional environmental data through multi-layer neural network structures [5], achieving breakthroughs in tasks such as multi-pollutant concentration prediction and pollution source identification. For instance, the SOPiNet model has realized synergistic inversion of PM_2.5_ and O_3_, improving accuracy by approximately 9% compared to traditional methods [6]. However, the “black-box” nature of deep learning models lacks traceability in decision-making processes [7]. In pollutant concentration prediction, models cannot clearly distinguish the contribution weights of environmental factors versus emission sources, severely limiting their application in policy-making and engineering practice [8].

By integrating explainable algorithms such as SHapley Additive exPlanations (SHAP) with physical mechanism constraints, Explainable Deep Learning (XDL) achieves dual interpretability of both model prediction outputs and underlying logic. (Interpretability denotes the property of a model that allows its internal logic, decision-making process, or feature contributions to be understood and verified by humans; explainability is synonymous with this definition.) Dual interpretability offers an alternative analytical approach for understanding model decision-making processes and enhancing the credibility of prediction results. For instance, Cremades et al. [9] demonstrated the potential of XDL in identifying critical physical regions in turbulence research, suggesting its promising application value in environmental governance scenarios.

As early as the late 20th century, the field of computer science initiated preliminary explorations into XDL. In 1980, Fukushima et al. [10] developed an artificial neural network named Neocognition. This network employed a hierarchical multi-layer design to perform recognition learning on computers. Due to its limited number of layers and fixed learning process, it exhibited rudimentary interpretability. In the early 21st century, researchers began integrating knowledge information to directly construct XDL models. Prior knowledge served as model input, while logical rules guided the learning process, significantly enhancing classification performance and interpretability [11]. This laid the foundation for XDL’s cross-domain applications. Widespread adoption in environmental science emerged around 2020, initially focusing on interpretable predictions for single pollutants [12]. Recent research has expanded toward multi-pollutant collaborative modeling. In 2023, a German research team extended the Layerwise Relevance Propagation (LRP) interpretability technique to Gated Recurrent Unit (GRU) neural networks. They analyzed key drivers for predicting concentrations of four pollutants—PM_10_, NO, NO_2_, and O_3_—and validated that simplifying the model by removing the encoder improved computational efficiency [13]. That same year, in the water environment domain, a Chinese research team constructed an explainable deep learning model integrating meteorological, land use, and socioeconomic variables. Using the SHAP method to identify dominant influencing factors, they predicted spatiotemporal changes in six river water quality parameters, providing scientific support for large-scale watershed water quality management [14]. In 2025, Yan et al. [15] proposed a multi-task deep neural network that simultaneously predicted four core water quality indicators—including the permanganate index—at multiple monitoring sites in Dianchi Lake based on inflow river data. SHAP analysis identified pollution contribution weights for each inflow river, achieving prediction accuracy improvements of up to 56.3% over traditional models and single-task deep learning models.

This study addresses a key question: Given the complex nonlinear interactions among multiple pollutants, how can XDL move beyond correlation-based predictions toward causal interpretation, thereby overcoming the low trust in black-box models? The review hypothesis of this paper is as follows: despite the distinct physical properties of atmospheric and aquatic media, XDL follows a unified technical logical framework in the coordinated control of multiple pollutants, namely “physical mechanism guidance combined with post hoc feature attribution”. A comparative study of these two media can reveal the universality and specificity of the XDL framework in applications across different environmental media.

This paper reviews the core scientific issues in synergistic multi-pollutant control and the technical framework of XDL. Focusing on two typical media—atmosphere and water bodies—it details the application progress of XDL in synergistic monitoring, source apportionment, mechanism analysis, and regulatory optimization. Current research bottlenecks are analyzed from three dimensions: modeling, data, and application. Aligning with disciplinary trends, a tripartite development pathway integrating “algorithm–mechanism–engineering” is proposed.

**Figure 1:**
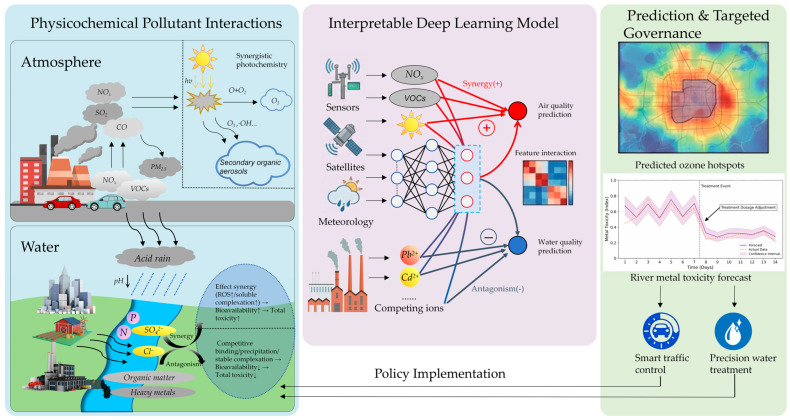
Technical framework of XDL application in multi-pollutant synergistic control.

The technical framework of XDL for multi-pollutant synergistic control is systematically illustrated in Figure 1, which takes the physicochemical interactions of pollutants as the core input, constructs an interpretable deep learning model as the technical core, and clarifies the synergistic/antagonistic response characteristics of typical atmospheric and aquatic media to multi-pollutants; the framework further links model output to practical governance applications such as air quality prediction, river metal toxicity forecast and precision water treatment, forming a complete technical chain from pollutant mechanism analysis to intelligent governance implementation.

## 2. Review Methodology

To systematically review the research progress of XDL in the synergistic governance of multiple pollutants, this study follows the principles of systematic literature review and refers to the PRISMA framework to ensure transparency and reproducibility. The search databases include Web of Science, Scopus, Google Scholar, and China National Knowledge Infrastructure (CNKI), covering the period from January 1980 to December 2025. The search keywords related to deep learning include: explainable deep learning, XDL; explainable AI, XAI; SHAP; interpretable deep learning; interpretable machine learning; LIME; and attention mechanism. Pollution-related search keywords include: multi-pollutant, synergistic governance, air pollution, water quality, PM_2.5_, O_3_, heavy metals, and organics. The inclusion criteria for the core literature are as follows: (a) the study involves both deep learning models and interpretability methods; (b) these models and methods are applied to synergistic monitoring, source apportionment, formation mechanism analysis, or regulation optimization of multiple pollutants in atmospheric or aquatic environmental media; (c) the study clearly provides model interpretation methods (e.g., SHAP, LRP, PDP) along with their quantitative or qualitative results. The exclusion criteria are: (a) studies focusing on a single pollutant without discussing synergistic or antagonistic effects with other pollutants; (b) studies that do not mention any interpretability method. In terms of defining the research scope, this paper focuses on two typical fluid media: the atmosphere and water bodies. Research on the coordinated control of multiple pollutants in these two media is the most mature, with sufficient literature support to enable a systematic horizontal comparison. Meanwhile, both media involve fluid dynamics and complex chemical reaction kinetics, which facilitate the investigation of XDL performance under physical consistency constraints. Therefore, this paper additionally excludes the fields of soil remediation and solid waste management, which are still in the initial stage of research, so as to ensure a focused discussion on the in-depth application of core algorithms and their transferability across different media.

Following the aforementioned methodology, an initial search yielded 1247 relevant articles. After duplicate removal, title and abstract screening, and full-text critical review, 95 articles were finally included for systematic analysis. This review adheres to a non-comparative principle when compiling the prediction accuracy of individual studies. Owing to significant differences in the datasets, spatiotemporal scales, validation strategies and data preprocessing methods adopted across various studies, the reported accuracy metrics (e.g., R^2^, RMSE) and their absolute values across the literature are not directly comparable in a cross-study manner. Therefore, in the tables and analyses presented below, the purpose of reporting these metrics is to demonstrate the performance levels claimed by each study itself or summarized from the literature in this review, as well as their relative merits in comparisons between different models within the same study—rather than conducting a cross-literature accuracy ranking of different studies. It is important to note that the validation strategies (e.g., temporal cross-validation, spatial cross-validation) vary greatly across studies, making it inappropriate to directly compare accuracy metrics across the literature. Meanwhile, R^2^ is susceptible to the variance of data and unable to reflect systematic biases, while RMSE and MAE are constrained by dimensions and value ranges and sensitive to large errors. For this reason, in addition to point estimation metrics, the physical consistency, uncertainty quantification and performance of models under extreme events are equally critical for synergistic governance decision-making. The processes of literature screening and information extraction for this study were independently conducted and cross-checked by Jie Hu and Jing Hua. Any discrepancies arising during the process were resolved through discussion or third-party arbitration.

## 3. Core Scientific Issues and Technical Requirements for Multi-Pollutant Management

### 3.1. Complexity Characteristics of Multi-Pollutant Management

The complexity of multi-pollutant synergistic management manifests across three levels: pollutant characteristics, environmental behavior, and management processes [16]. In the atmosphere, PM_2.5_ and O_3_ exhibit a “see-saw” effect; in water bodies, heavy metals form complexes with humic acids to enhance toxicity; and in soil, antibiotics and heavy metals mutually influence bioavailability. These synergistic or antagonistic interactions significantly increase management difficulty [17]. At the environmental behavior level, pollutant migration and transformation exhibit strong spatiotemporal heterogeneity. For instance, peak concentrations of nitrogen and phosphorus in urban black and odorous water bodies are temporally and spatially misaligned with heavy metal peaks, while atmospheric pollutant transport pathways vary significantly under different meteorological conditions [18,19]. Remediation processes often involve trade-offs: reducing SO_2_ can raise O_3_ levels, and chemical flocculation for heavy metals may create secondary pollution. This underscores the urgent need for systematic synergistic control strategies [20,21].

### 3.2. Limitations of Traditional Control Technologies

XDL, with its ternary structure of “deep learning architecture + explainable module + physical constraint”, has initially demonstrated the potential to meet the technical requirements for synergistic control of multi-pollutants. However, physicochemical models—built upon fundamental laws of fluid dynamics and thermodynamics, such as the WRF-Chem model for atmospheric applications and the EFDC model for water bodies—rely heavily on comprehensive emission inventories and parameter settings. They exhibit substantial errors under complex land surface conditions and incur high computational costs, making them ill-suited for real-time regulatory demands [22,23]. Traditional machine learning methods in data-driven models, such as Random Forests, offer computational efficiency but lack sufficient feature extraction capabilities for high-dimensional spatiotemporal data, failing to capture deep correlations between pollutants [24].

More critically, both model types suffer from insufficient interpretability. While physicochemical models offer theoretical interpretability, their complex parameterization processes make their actual decision-making logic difficult to trace. Feature importance analysis in traditional machine learning models is predominantly global statistical, failing to provide personalized explanations for local samples and making it harder to quantify nonlinear interactions between pollutants. These limitations hinder traditional technologies from supporting the precise, differentiated requirements of multi-pollutant synergistic management.

### 3.3. Technical Suitability of XDL

XDL aligns with multi-pollutant synergistic management requirements through its tripartite structure: “deep learning architecture + interpretability module + physical constraints.” At the feature extraction level, deep learning components like convolutional layers and attention mechanisms effectively capture spatiotemporal correlations among pollutants. For instance, the SOPiNet model employs multi-head attention to uncover spatiotemporal coupling between PM_2.5_ and O_3_ [6]. At the interpretability level, methods like Gradient Boosting and SHAP enable multi-scale explanations from global to local scales. For instance, the XGBoost–SHAP model quantifies the contribution of pollutants like NO_2_ and CO to PM_2.5_ [25]. At the mechanism integration level, incorporating physical regularization terms into the loss function allows models to train with mechanisms like diffusion equations and adsorption–desorption kinetics, enhancing the scientific rigor of interpretable results [26].

Compared to traditional techniques, XDL achieves the unity of “precise prediction” and “clear explanation,” with its technical advantages primarily manifested in three aspects: (1) Nonlinear interaction analysis capability—used to quantify concentration-dependent antagonistic effects among multiple components; (2) Spatiotemporal dynamic explanation capability—enabling tracking and analysis of real-time changes in pollutant transport pathways and source contributions; (3) Multi-objective synergistic optimization capability—enabling quantitative analysis of the contribution each effect makes to the final pollutant load reduction through the optimization of multiple overlapping effects.

## 4. Core Theory and Technical Framework of XDL

### 4.1. Essence and Classification of XDL

XDL refers to a technical system that maintains the high-precision predictive capabilities of deep learning while enabling the decision-making process, feature contributions, and underlying logic of the model to be understandable, traceable, and verifiable through specific algorithmic or model architecture design [27]. Based on different interpretation approaches, it can be categorized into intrinsically interpretable models and post hoc interpretation methods.

Intrinsic interpretable models enhance model interpretability by simplifying network structures or introducing prior constraints, mainly including sparse neural networks, attention mechanism networks, and physics-informed neural networks. Sparse neural networks achieve automatic feature selection via L1 regularization, reducing interference from redundant variables [28]. Attention-based networks visually highlight contributions from critical spatio-temporal nodes through weight distribution. For instance, the dual-task attention layer in SOPiNet emphasizes key regions for synergistic PM_2.5_ and O_3_ inversion [6]. Physically inspired neural networks (PINNs) incorporate physical equations as soft constraints within loss functions, achieving unified prediction accuracy and physical consistency in atmospheric pollutant dispersion simulations [29].

Post hoc methods preserve the model structure and achieve interpretability by analyzing input–output relationships or internal parameters. They fall into two categories: model-agnostic and model-specific. Model-agnostic methods are applicable to diverse deep learning models. For instance, SHAP quantifies each feature’s contribution to predictions based on game theory principles and has been widely applied to elucidate the driving mechanisms of pollutants like PM_2.5_ and O_3_ [30]. LIME constructs interpretable models through local linear approximation, enabling visualization of decision bases for individual samples [31]. Model-specific methods target particular network architectures, such as the Class Activation Map (CAM) for CNNs, which locates key spatial regions influencing pollutant concentrations [32], and gate weight analysis for LSTMs, which identifies critical temporal nodes [33].

### 4.2. Principles and Characteristics of Core Interpretation Algorithms

Among commonly used interpretable algorithms for multi-pollutant collaborative governance, SHAP stands out. In air pollution forecasting, SHAP commands the highest adoption rate at 46.4% among all interpretable methods [34]. The core of SHAP is the Shapley value, which is used to measure the fair contribution of an individual participant to the total value in a cooperative game and serves as the theoretical origin of SHAP [35]. Derived from game theory, this value is applied to the fair allocation of contributions among individual “players” within a “coalition”. In environmental models, the “coalition” refers to the set composed of all input features, the “players” are individual features, and the “coalition value” corresponds to the model’s predicted concentration of a certain pollutant [36]. The SHAP value of an individual feature quantifies the average marginal contribution of the presence or absence of that feature to the final predicted value. Its theoretical formula is given as follows:

(1) $$\phi_{i}=\sum_{S\subseteq \{1,\ldots ,n\}\setminus \{i\}}\frac{|S|!(n-|S|-1)!}{n!}\left[f(S\cup \{i\})-f(S)\right]$$

where $\phi_{i}$ denotes the SHAP value of feature i. In environmental applications, it represents the final contribution of a specific driving factor (e.g., atmospheric NO_2_ concentration or water temperature in aquatic systems) to the model prediction, with units consistent with those of the predicted target (such as μg/m^3^ or mg/L). A positive value indicates that the feature increases the predicted value (a “deteriorating” contribution), whereas a negative value means it reduces the predicted value (an “improving” contribution). n is the set of all input features. For instance, in predicting riverine total phosphorus, n may include 10 features such as water temperature, pH, discharge, upstream total nitrogen concentration, and land-use type. S represents a subset of features excluding feature i. f(S) is the model output predicted using only the feature subset S. This is critical for interpreting the formula: it represents the model prediction based on other available information “without” feature i. The term f(S∪{i})−f(S) is the change in the model prediction after incorporating feature i. From an environmental perspective, this directly quantifies the marginal effect of the driving factor (e.g., a 5 °C rise in water temperature) on the target pollutant concentration across all possible feature combinations. The weighting coefficient $\frac{|S|!(n-|S|-1)!}{n!}$ ensures that contributions from all subsets are fairly allocated.

Equation (1) essentially computes the “average marginal contribution” of a feature across all possible scenarios. This is essential for disentangling complex nonlinear antagonistic and synergistic interactions in multi-pollutant systems. For example, in the “seesaw effect” between atmospheric O_3_ and PM_2.5_, the SHAP value of VOCs (ΦVOCs) can quantify their net contribution to O_3_ formation under all combinations of meteorological conditions and NO_x_ concentrations [37]. If ΦVOCs is significantly positive under high-temperature and low-NO_x_ conditions, the model “explains” that VOCs act as the key driver of O_3_ formation under such circumstances [38].

SHAP supports combining global and local explanations, but computational complexity grows exponentially with the number of features n. Direct calculation using the general formula has O(2n) complexity, making it impractical for high-dimensional data. In multi-pollutant studies, feature selection techniques must be integrated for optimization [39]. In practice, efficient solutions are often achieved using algorithms like DeepSHAP, KernelSHAP, and TreeSHAP.

(2) $$\phi_{i}=(x_{i}-x\prime_{i})\cdot \frac{\partial f(x)}{\partial x_{i}}{|}_{x=x\prime }$$

(3) $$\phi =\arg\underset{\phi }{\min}\sum_{S\subseteq N}w(S){\left(f(S)-\sum_{i\in S}\phi_{i}\right)}^{2}$$

Equations (2) and (3) represent the formulations of DeepSHAP and KernelSHAP, respectively. $x_{i}$ denotes the feature value of the current sample (e.g., the measured PM_2.5_ concentration at a specific time), and $x\prime_{i}$ is the baseline reference value (e.g., the mean PM_2.5_ concentration across all sampling times). $\frac{\partial f(x)}{\partial x_{i}}$ represents the gradient of the model with respect to feature i at the given sample, reflecting the local sensitivity of the model’s decision boundary to feature i at that point. The full DeepSHAP formula can be interpreted as follows: the contribution of feature i is determined by the deviation of its value $x_{i}$ from the baseline, multiplied by the model’s local sensitivity to that feature. KernelSHAP is a model-agnostic approximation approach that estimates SHAP values by solving a weighted linear least-squares problem. This formula aims to find an optimal set of contribution values $\phi_{i}$ such that, for any feature subset S, the sum of contributions of the features within the subset approximates the actual model output f(S) using that subset as closely as possible. The weight w(S) ensures that the errors from subsets of different sizes are properly balanced.

DeepSHAP is specifically designed for deep learning models and is well-suited for explaining individual pollution events (local interpretability) [40]. The “black-box” nature of KernelSHAP makes it particularly applicable for comparing the attribution consistency of different models for the same environmental problem [41]. TreeSHAP is an optimization for decision trees, reducing computational complexity and applicable to tree models like XGBoost and RF [42].

In practice, SHAP is often combined with other algorithms based on research objectives. Like SHAP, Partial Dependency Plots (PDPs) are pattern-agnostic methods. By fixing other features, they plot the relationship curve between the target feature and prediction outcomes, visually demonstrating marginal effects [43]. This approach identifies key pollution features, enhances the researcher’s understanding, and makes machine learning results more accessible to non-specialists. In predicting nitrogen and phosphorus levels in water bodies, PDPs clearly reveal the abrupt increase in phosphorus concentration when water temperature exceeds 18 °C, providing evidence for eutrophication early warning [44]. The advantage of the PDP method lies in its intuitive and easy-to-understand interpretation. Its disadvantage is the inability to capture interactions between features, which can lead to misleading results when strongly correlated features are present [45].

### 4.3. XDL’s Technology Integration Framework

Combined with the technical framework of XDL in multi-pollutant synergistic control shown in Figure 1, the integrated application of XDL technology can be decomposed into a multi-layered technical system with clear logical links, from the bottom-up data preprocessing and model construction to the top-down interpretability analysis and governance application output.

The data preprocessing layer integrates and enhances multi-source data. To address the prevalent issue of missing values in environmental data, Generative Adversarial Networks (GANs) can be employed for data augmentation. Peter et al. [46] employed GANs to learn correlations in input layer data for precise missing value imputation. This was combined with a hierarchical attention mechanism and bidirectional LSTM (BiLSTM) to construct a hybrid prediction model. Applied to India’s 2015–2020 air quality data (including PM_2.5_), it achieved low-error predictions. Metrics such as MAE, RMSE, and MAPE significantly outperformed existing models like linear regression and LSTM, substantially enhancing time series prediction accuracy.

The model construction layer serves as the core execution unit, employing a collaborative hybrid architecture integrating foundational networks with physical constraints. The foundational network is selected based on task type: CNN + attention mechanism (SOPiNet model) for collaborative inversion tasks, GR-BiLSTM model for time-series prediction tasks, and the multi-task learning framework [6,47,48] for source apportionment tasks. Physical constraints can embed physical operators within the network structure. For instance, the atmospheric diffusion Advection–Diffusion–Reaction (ADR) equation is incorporated as a specialized layer into CNNs to form ADRNet. This enables rapid non-local information transfer via semi-Lagrangian pushdown operators, significantly enhancing the accuracy and long-term stability of spatiotemporal sequence predictions [49]. Alternatively, physical regularization terms can be incorporated into the loss function. For instance, the PINN framework was employed to develop a subsaturated soil flow simulation method by embedding the Richards equation (RRE), which describes flow dynamics, into the model’s loss function. This ensures predictions align with fundamental flow principles like mass conservation [50].

The interpretability layer enables multidimensional result analysis. Global explanations identify key drivers through SHAP value ranking. For instance, the LightGBM-SHAP model revealed secondary particulate matter (31.1%) and biomass burning (25.1%) as primary contributors to metropolitan-scale PM_2.5_ [51]. Kanayankottupoyil et al. [52] developed a GRU-LSTM hybrid deep learning model integrated with SHAP analysis. By utilizing hourly precursor and meteorological data, they achieved precise ground-level O_3_ concentration predictions for North Texas (R^2^ ≈ 0.97, IoA > 0.96) while quantifying key factors influencing O_3_ formation. Local explanations analyze decision logic for individual samples using methods like LIME and IG. A V et al. [53] proposed ensemble regression models based on gradient boosting, XGBoost, and CatBoost, combined with LIME local explanations to forecast hourly PM_2.5_ concentrations in Bengaluru.

The application output layer translates interpretive results into governance decisions. Source apportionment reports clarify the contribution ratios and spatial distribution of various pollution sources; control schemes formulate differentiated emission reduction strategies based on sensitivity analysis. Chen et al. [54] developed differentiated NMHC emission reduction plans for counties and cities in Taiwan, China, using CMAQ-DDM sensitivity analysis as the core and combining it with linear programming. Effect prediction simulates the governance outcomes of different schemes through perturbation analysis. Hong-Zhao et al. [55] developed the GR-BILSTM deep learning fusion model, which quantifies the nonlinear relationship between industrial emissions and PM_2.5_ concentrations via perturbation analysis, achieving a coefficient of determination R^2^ = 0.83.

### 4.4. Boundaries and Challenges of Physics–Data Fusion: When Interpretability Encounters Physical Inconsistencies

The above technical framework implicitly relies on a critical assumption: that physical mechanisms and monitoring data are inherently compatible, and the role of interpretable models is merely to “uncover” their consistency. However, in real-world scenarios of multi-pollutant co-governance, conflicts between physical laws and data are the norm rather than the exception, yet existing XDL methods lack a systematic critical and handling mechanism for such discrepancies.

Although PINNs excel in idealized inverse problems of partial differential equations (PDEs), they suffer from three fundamental limitations in multi-pollutant co-governance. First, spectral bias: PINNs tend to prioritize learning low-frequency, large-scale concentration distributions while struggling to fit high-frequency signals such as pollutant concentration fronts and sudden emission pulses, leading to systematic over-smoothing of prediction biases for heavy pollution events [56]. Second, multi-scale stiffness: When fast reactions (second-scale) and slow transport (hour-scale) coexist in atmospheric chemistry, the magnitudes of individual physical terms in the PINN loss function can differ by several orders of magnitude, making gradient optimization highly prone to local optima [57]. Third, interpretability paradox: PINNs enhance the physical consistency of predictions via physical constraints, yet they themselves remain “black boxes”; when physical residual terms conflict with data-fitting terms, mainstream interpretability methods such as SHAP and LIME cannot distinguish whether model bias stems from data noise, network architecture, or the inappropriateness of the physical equations themselves [58].

The core dilemma of physics–data fusion can be summarized into three typical categories of conflicts. First, incomplete physical knowledge: For instance, the synergistic transport of combined antibiotic–heavy metal pollutants in water bodies lacks a closed-form PDE description [59]; enforcing mass conservation formulations in such cases introduces “precisely wrong” results. Under these circumstances, extrapolative interpretations by the model outside physically constrained regions are entirely unreliable. Second, parametrization controversies: Multiple competing parametrization schemes exist for the multiphase chemical mechanisms of secondary organic aerosol (SOA) formation in the atmosphere, leading PINN constraints to diverge in opposite directions across different schemes. Existing XDL studies rarely address the sensitivity of SHAP attribution results to the choice of physical equations. Third, direct contradictions between observations and physical laws: For example, monitoring data may show an increase in local PM_2.5_ concentrations accompanied by a decline in precursor emissions, contradicting expectations from diffusion equations. In such cases, SHAP would attribute this discrepancy to “unobserved variables”, while PINNs would enforce fitting to the physical equations, yielding drastically different interpretations—yet the model itself cannot output a confidence level indicating that “the current interpretation is unreliable”.

Existing XDL frameworks exhibit asymmetric interpretability vulnerability when physical constraints break down. Overweighting physical regularization terms causes the model to sacrifice accuracy at monitoring sites to maintain global physical consistency, resulting in missed detections of local heavy pollution [60]; underweighting these terms reduces the model to a purely data-driven approach, whose interpretations align statistically with SHAP but violate basic physical intuition [61]. A more fundamental issue is that current XDL lacks a physical conflict detection mechanism: when predictions deviate significantly from physical residual terms, the model cannot distinguish between data anomalies, erroneous equations, or missing processes, nor can it provide actionable warnings to decision-makers.

Breaking through these bottlenecks requires reconstructing the physics-fusion paradigm of XDL. This entails shifting from “hard constraints” to “probabilistic constraints”, representing physical laws as prior distributions with uncertainty rather than deterministic equalities; developing a physical conflict interpretability module that enables the model to output a “data–physics consistency index” alongside predictions; and establishing a hierarchical physics-fusion strategy—applying strong constraints to well-established mechanisms such as mass conservation, weak regularization to contentious mechanisms such as SOA formation, and retaining pure data-driven modeling for unknown processes.

## 5. Application Progress of XDL in Multi-Pollutant Control of Atmospheric Environment

### 5.1. Analysis of Driving Factors for Multiple Pollutants Based on SHAP and Tree Models

The primary challenge in the synergistic control of multiple atmospheric pollutants lies in clarifying the complex nonlinear interactions among PM_2.5_, O_3_ and their precursors. The combination of SHAP-centered interpretable frameworks and tree-based models (XGBoost, Random Forest) has become a mainstream paradigm for analyzing the driving factors of multiple pollutants. As shown in Table 1, in applications within the atmospheric field, the combined architecture of “tree-based models + SHAP” covers diverse scenarios ranging from the synergistic inversion of PM_2.5_ and O_3_ and source apportionment of HONO to the prediction of health oxidative potential, verifying the wide applicability of this paradigm in the synergistic control of multiple pollutants. Numerous studies have demonstrated that such methods can effectively distinguish the differential contributions of meteorological factors and emission sources to pollutant concentrations. For instance, a study on O_3_ pollution in Hangzhou found that meteorological factors accounted for approximately 58% of O_3_ concentration variability, with relative humidity and ultraviolet radiation as the dominant driving factors, while alkenes and oxygenated volatile organic compounds (VOCs) dominated the chemical effects [62]. Similarly, in a PM_2.5_ study in the Urumqi–Changji–Shihezi region, SHAP analysis revealed that surface pressure and O_x_ (NO_2_ + O_3_) together explained over 70% of the meteorological variability, and the dominant factor shifted from pressure in 2019 to Ox in 2021 [63]. These findings indicate that interpretable models can not only quantify the contribution shares of various factors but also capture the temporal evolution of dominant mechanisms.

Nevertheless, existing studies have notable methodological limitations. First, the interpretation of SHAP values is highly dependent on the quality and completeness of input features; when strong correlations exist between features (e.g., temperature and seasonal encoding), traditional univariate partial dependence plots may yield misleading conclusions. A study on PM_2.5_ estimation in China explicitly pointed out that when temperature is highly correlated with temporal encoding, one-dimensional partial dependence plots produce a spurious relationship of “rising temperature leading to increased PM_2.5_” that contradicts physical reality, whereas two-dimensional partial dependence plots reveal the true negative correlation [69]. Second, most studies only report feature importance rankings, with few exploring the attribution stability of models across different concentration intervals (e.g., light and heavy pollution). A study in Zibo showed that the contribution of NH_4_^+^ to PM_2.5_ can differ by several times between clean and polluted periods, and such concentration-dependent interpretive variability has not received systematic attention [64].

A more fundamental issue is that current attribution analyses mostly remain at the level of statistical correlation, lacking rigorous testing of causal mechanisms. Although SHAP can quantify “how Y changes as X changes”, it cannot answer the question of “whether changing X will inevitably lead to changes in Y” that policymakers truly care about. To address this, some studies have attempted to compensate for this deficiency by incorporating physical constraints, such as introducing residual terms of the continuity equation into the loss function to ensure that deep learning model predictions comply with the law of mass conservation [68]. This physics-informed fusion approach provides a feasible path from statistical correlation to causal inference, but its integration into the SHAP interpretation framework is still in its infancy.

### 5.2. Physics-Informed Deep Learning Methods for Synergistic Control

To overcome the shortcoming of single-pollutant modeling that ignores interactions between pollutants, multi-task learning and physics-informed fusion models have emerged. The core hypothesis of these methods is that PM_2.5_ and O_3_ share common precursors (NO_x_, VOCs) and meteorological conditions, and joint modeling can improve overall estimation accuracy and computational efficiency. Experiments have verified that the deep network (SOPiNet) for simultaneous inversion of PM_2.5_ and O_3_ improves the R^2^ value by 6 percentage points compared with single-task models, and the gain of joint training becomes more pronounced when the data missing rate of a certain pollutant exceeds 20% [6]. Another study further expanded the multi-task framework by using a self-attention mechanism to differentially process the effects of shared features on different pollutants, and introducing the physical constraint that PM_2.5_ concentration must be lower than PM_10_, achieving R^2^ values of 0.92 (O_3_), 0.90 (PM_2.5_) and 0.87 (PM_10_) for joint estimation [66]. These results demonstrate that knowledge-guided model design—including categorical encoding of input features, construction of interaction modules, and embedding of physical constraints—is key to enhancing the performance of synergistic estimation of multiple pollutants.

However, there are significant divergences in the strategic choices of physics-informed fusion. One approach favors a “soft constraint” strategy, which adds physical residual terms to the loss function as regularization terms. For example, a graph convolutional network-based air quality estimation model introduces residual loss from the continuity partial differential equation, ensuring mass conservation in predictions at unobserved spatiotemporal locations and improving the R^2^ value by 11–22% in out-of-site extrapolation tests compared with purely data-driven methods [68]. The relative percentage of improvement against the same benchmark serves as a valid metric for evaluating the contributions of different technical strategies and holds greater scientific significance than the comparison of absolute R^2^ values across studies. Another approach adopts a “hard constraint” strategy, directly designing network architectures to enforce physical laws, such as ensuring atomic conservation in chemical mechanism simulations [70]. The two strategies have respective advantages and disadvantages: soft constraints offer high flexibility but insufficient guarantee of physical consistency, while hard constraints provide high physical fidelity but limited model generalization. Current studies have not reached a consensus on which strategy is more suitable for the synergistic control of multiple pollutants, which, to some extent, reflects that the theoretical foundation for integrating domain knowledge and data-driven methods remains weak.

Notably, as shown in Table 1, existing physics-informed models mainly focus on the “pairwise synergy” of PM_2.5_ and O_3,_ while research on joint estimation of six conventional pollutants (including CO, SO_2_, and NO_2_) is relatively insufficient. Although some studies have attempted to estimate multiple pollutants simultaneously, their interpretive analyses often stay at the level of “which factors are important” and fail to reveal the interaction mechanisms between pollutants—for example, does elevated CO concentration affect O_3_ formation directly or indirectly by influencing OH radical concentration? The root cause of this limitation is that current physics embedding is mostly based on universal physical laws, such as mass conservation, and there is still a lack of systematic methodology for designing constraints targeting chemical reaction mechanisms between specific pollutants.

### 5.3. Source Apportionment and Spatiotemporal Attribution Fused with Multi-Source Data

The application of XDL in atmospheric pollution source apportionment is evolving from traditional “source contribution quantification” to “source–receptor dynamic relationship identification”. The combination of traditional Positive Matrix Factorization (PMF) and machine learning enables researchers to identify pollution sources while quantifying the nonlinear contributions of each source to health effect indicators (e.g., oxidative potential). An XGBoost–SHAP framework applied to a haze event in Qingdao found that water-soluble organic carbon and K^+^ contributed 25.6% and 22.3% to PM_2.5_ oxidative potential, respectively, whereas traditional PMF models significantly underestimated contributions from the same sources [67,71]. This discrepancy suggests that source apportionment under linear assumptions may systematically underestimate the health risks of certain toxic components. Similarly, a study on the PM_2.5_ sensor network in Dhaka used generalized additive models to separate the effects of boundary layer height, long-range transport and local wind fields, finding that air mass transport from the Indo-Gangetic Plain can increase PM_2.5_ concentrations by 40 μg/m^3^, while local road emissions exert an impact of approximately ±20% [72].

However, source apportionment research faces the severe challenge of spatiotemporal scale mismatch. There is an essential difference between satellite-retrieved pollutant column concentrations and ground monitoring data: the former reflects the integrated atmospheric column, while the latter represents near-surface concentrations, with their relationship modulated by boundary layer height, aerosol vertical distribution and other factors. Although deep learning models can “learn” this mapping relationship from large datasets, their performance often degrades sharply when transferred to regions with vastly different meteorological conditions. A study of six typical cities in China showed that PM_2.5_ estimation based on the Convtrans model achieved an R^2^ of 0.92 within training cities but dropped to 0.85 in spatial cross-validation, with this decline varying significantly across cities [65]. This heterogeneous generalization ability essentially stems from overfitting of training data to specific meteorological-emission combinations, and interpretable methods have failed to effectively reveal under what conditions the model will fail.

A more fundamental problem is that current interpretable attributions are mostly based on statistical correlations, making it difficult to distinguish the true contributions of local emissions and regional transport. Although the combination of trajectory clustering and SHAP can identify concentration increments from specific transport pathways, this method implies an assumption—that samples with similar backward trajectories have similar source contributions—which often does not hold in the real atmosphere, as emission source intensities change dynamically over time. In response, some researchers have introduced a “meteorology-removing” method to strip the influence of meteorological conditions via random resampling of meteorological variables, thereby isolating the net contribution of emissions [63]. The application of this method during the COVID-19 lockdown period showed that reduced emissions explained the majority of PM_2.5_ declines in the Urumqi–Changji–Shihezi region, while changes in meteorological contributions accounted for less than 5%. This finding validates the effectiveness of the “meteorology-removal + interpretable model” framework in event attribution, but its treatment of temporal autocorrelation and spatial heterogeneity still involves methodological simplifications that require further improvement.

## 6. Application Progress of XDL in Multi-Pollutant Control of Aquatic Environment

### 6.1. Paradigm of Pollution Attribution Analysis Based on SHAP and Tree Models

The combination of the SHAP-centered interpretable framework and tree-based models (Random Forest, XGBoost) has become the mainstream paradigm for attribution analysis of multiple water pollutants. As shown in Table 2, the core advantage of such methods lies in their ability to quantify the differential contributions of meteorological, land-use, and socioeconomic factors to single or multiple water quality indicators, while overcoming the insufficient fitting of complex nonlinear relationships by traditional linear models. Studies on river water quality prediction have shown that after incorporating land-use and socioeconomic variables into the model, the R^2^ for total nitrogen (TN) and total phosphorus (TP) prediction increased from 0.17–0.39 to 0.58–0.60, an improvement of up to 230%. Further SHAP analysis identified cultivated land area, forest cover rate, and air temperature as the most important factors affecting dissolved oxygen (DO), TN, and TP, respectively [73]. Similarly, in applications in the Yangtze River Basin, SHAP revealed the positive driving effects of slope and population density on TP, as well as the beneficial contribution of forests to DO—findings highly consistent with watershed hydrological theory [74]. These studies demonstrate that XDL not only improves prediction accuracy but also transforms models from “black boxes” into verifiable tools for scientific hypothesis testing.

Nevertheless, current SHAP-based attribution studies suffer from two noteworthy methodological limitations. First, the interpretability of SHAP values heavily relies on the independence of input features and data quality. When spatial collinearity exists among land-use, socioeconomic, and meteorological variables (e.g., high-GDP regions often coincide with high built-up area ratios), SHAP estimates of individual feature contributions may be biased. A study in the Taihu Basin found that although TN and TP were identified as mutually influential key factors via SHAP analysis, their causal direction (whether TN drives TP or vice versa) could not be determined by SHAP values alone and required cross-validation with high-frequency water quality monitoring data [75]. Furthermore, most studies only report global feature importance rankings while ignoring the dynamic evolution of driving factors under different hydrological scenarios (e.g., wet and dry seasons). Research in the Laoshan River Basin shows that the contribution of turbidity (TU) to TP prediction rises significantly in the wet season due to enhanced non-point source pollution input via runoff, yet this seasonal pattern is nearly flattened in annual average SHAP values [77].

A more fundamental issue is that the attribution provided by SHAP is essentially “correlational” rather than “causal”. Although researchers often interpret high SHAP values as “driving factors”, the model only captures statistical correlations. For instance, subwatersheds with high forest cover often exhibit high DO, which may stem from forest interception of pollutants or spatial separation between forested areas and industrial pollution sources. To address this shortcoming, some studies have attempted to introduce physical constraints: outputs from process-based models such as SWAT (e.g., nitrate load contributed by lateral flow) are fed as features into XGBoost, allowing SHAP attributions to gain process-based support [85]. While this “process–data fusion” approach is directionally correct, it essentially remains an improvement at the feature engineering level and has not yet achieved a methodological leap from statistical correlation to causal inference.

### 6.2. Physics-Informed and Multi-Task Learning Frameworks for Synergistic Prediction of Multiple Pollutants

To address the limitation of single-pollutant modeling that overlooks interactions among pollutants, multi-task learning and physics-informed fusion models have emerged as a new research focus. The core hypothesis of these methods is that different water quality parameters share common environmental drivers and transformation pathways, and joint modeling can improve overall estimation accuracy and computational efficiency. Applications of a hybrid CNN-LSTM-XGBoost model across multiple watersheds demonstrate that using multiple water quality and environmental time-series features (including pH, turbidity, and DO) as joint inputs to predict water pollution levels increases classification accuracy from 88.7% to 94.6% compared with univariate models using only single pollution-related features. The misclassification rate for medium and high pollution levels is significantly reduced, and the MSE for numerical prediction drops to 0.011 [79]. Another study extended this approach by coupling CEEMDAN data decomposition with LSTM-Transformer, enabling the model to separately handle high-frequency fluctuations, medium-frequency seasonal variations, and low-frequency trend components in TP time series. The overall R^2^ reached 0.87, an improvement of 5 percentage points over the undecomposed LSTM model [77].

However, significant divergences exist in model design strategies for multi-task learning. The multi-pollutant joint prediction models listed in Table 2 reflect this divergence: the WD-LSTM-Transformer model using hard parameter sharing achieved an R^2^ exceeding 0.92 for both TN and TP predictions, whereas the ANN model with the same multi-output architecture only obtained an NSE of 0.68 in TN prediction. This indicates that hard sharing may lead to negative transfer between tasks when the driving mechanisms of different water quality parameters differ significantly. One approach favors “hard parameter sharing”, where all prediction tasks share a bottom-level feature extraction network and only diverge at the output layer. This design offers high parameter efficiency and strong generalization ability but may cause negative transfer between tasks when driving mechanisms differ substantially across water quality parameters (e.g., DO is strongly affected by water temperature, while turbidity is mainly controlled by runoff). A study in an urban watershed in southern Italy showed that a multi-output ANN model simultaneously predicting TSS, TN, and TP achieved an NSE of only 0.68 for TN, whereas a single-output model predicting TN alone reached 0.84 [80], indicating that multi-task learning may degrade prediction accuracy when task correlation is insufficient. Another approach adopts “soft parameter sharing” or task-specific attention mechanisms, allowing for different tasks to learn shared features while retaining task-specific representations. When applied in a reservoir watershed in Galicia, Spain, this design boosted the joint prediction R^2^ for five water quality indicators (DO, pH, TA, BOD_5_, and TN) to 0.9469, 0.8874, 0.8977, 0.9604, and 0.9138, respectively—an average improvement of 280% over traditional CNN-LSTM models. The tradeoff is a substantial increase in model parameters due to the integration of dual attention and cross-stitching components compared with the base CNN-LSTM model [78].

A more challenging issue is that the integration of multi-task learning frameworks with physics-informed constraints has yet to form a systematic methodology. The current mainstream practice is to use simulated outputs from process-based models (e.g., SWAT) as additional features for deep learning models, which essentially remains as feature engineering under the data-driven paradigm rather than genuine physical constraint embedding. For example, after feeding SWAT-simulated discharge, suspended solids (SS), TN, and other outputs as input features to LSTM, the RMSE for TP prediction decreased drastically (by 81.9% at the watershed outlet), yet no interpretability analysis was conducted. Although the inherent uncertainty of traditional SWAT parameter calibration was avoided, the overall uncertainty of the hybrid model was not eliminated through the combined design [84]. In contrast, embedding physical constraints directly into the loss function (e.g., mass conservation, mass balance) remains extremely rare in water pollutant prediction, in sharp contrast to practices in atmospheric research (e.g., synergistic estimation of air pollutants) [82]. The root cause of this gap is that the transport and transformation of water pollutants involve multiple processes, such as adsorption–desorption, biodegradation, and sedimentation–resuspension, whose parametric representations in physical equations remain highly controversial, depriving the “hard constraint” strategy of a reliable physical foundation.

### 6.3. Graph Neural Networks and Uncertainty Quantification for Small Samples and Spatial Heterogeneity

Faced with practical challenges such as sparse monitoring sites, severe data missingness, and strong watershed spatial heterogeneity, graph neural networks (GNNs) and Bayesian deep learning methods have demonstrated unique application potential. Unlike traditional CNN and LSTM, which assume that data lie on regular grids, GNNs directly model the spatial topological relationships between monitoring sites, treating sites as nodes and hydrological connectivity or spatial proximity as edges [86]. Research on groundwater pollution transport shows that an attention-based graph neural network (aGNN) achieved an R^2^ of 99% for pollutant concentration prediction when processing data from 51 irregularly distributed monitoring sites, significantly outperforming ConvLSTM (R^2^ = 90%) and DCRNN trained on the same data. Critically, aGNN can infer pollutant concentrations at unmonitored sites via inductive learning, with prediction errors controlled below 0.3 mg/L within 500 m of known sites, providing methodological support for optimizing monitoring network design [82]. However, graph structure construction is inherently subjective: edges defined by Euclidean distance cannot fully reflect actual hydrological connectivity, while edges based on watershed topology rely heavily on the resolution and accuracy of digital elevation models. This “graph structure uncertainty” has scarcely been quantified in existing studies.

Bayesian deep learning provides an alternative technical path for uncertainty quantification in data-sparse scenarios. Unlike traditional deep learning, which outputs point estimates, Bayesian neural networks (BNNs) treat network weights as probability distributions, enabling the simultaneous prediction of values and their confidence intervals. Research on mass flux prediction in DNAPL source zones shows that BNNs maintain high prediction accuracy when processing laboratory small-sample data, with 95% confidence intervals fully covering measured effluent concentrations. In contrast, traditional deterministic upscaling models not only yield higher prediction errors under the same data but also exhibit significantly larger biases at extreme concentrations and during complex dissolution stages [81]. Notably, uncertainty in BNNs primarily derives from the posterior distribution of model parameters rather than input feature uncertainty—yet the latter is often more critical in environmental monitoring (e.g., instrument errors, sampling time deviations). Current studies have not resolved the methodological challenge of unifying the quantification of these two types of uncertainty.

Data imbalance is particularly pronounced in the prediction of trace pollutants. Studies on concentration classification of watershed micropollutants (pesticides, pharmaceuticals) show that low-concentration samples (0–20th percentile) account for over 77.5% of data, while high-concentration samples make up only approximately 4%. Classification models trained on raw imbalanced data tend to favor low-concentration samples, leading to poor accuracy for high-concentration cases. After applying the SMOTE oversampling technique, the AUC of the XGBoost model for high-concentration samples improved by about 13 percentage points [76]. However, synthetic samples generated by SMOTE may introduce “spurious patterns” that violate physical laws [87]—for example, combinations of high pesticide concentrations and low rainfall that are environmentally implausible. This limitation arises because data augmentation methods only rebalance statistical distributions while ignoring physical constraints between variables in environmental systems. Some studies have attempted to embed prior knowledge from process models into data augmentation (e.g., generating samples only along groundwater recharge paths), but the associated computational cost is prohibitively high for widespread application in real-world monitoring networks.

## 7. Comparison of XDL Applications in Atmospheric and Aquatic Environments

As two typical fluid media, atmospheric and aquatic environments share the unified technical logic of “physical mechanism guidance + post-hoc feature attribution” in XDL applications, yet exhibit divergent technical approaches due to inherent differences in their physical properties and pollution process characteristics.

The two share three common features: both adopt the “tree model + SHAP” as the core attribution paradigm, both face the methodological bottleneck of transforming statistical correlation into causal inference, and both explore multi-task learning to capture the synergistic effects among pollutants.

The core differences are reflected in three dimensions. First, the depth of physical constraint embedding varies. The atmospheric field has formed a mature dual system of “soft constraints + hard constraints”; for instance, ADRNet directly integrates the advection–diffusion equation as a network layer. In contrast, the aquatic field remains at the stage of shallow fusion, where “outputs of mechanistic models are used as input features”, with almost no practical application of hard constraints. Although physical constraints are more deeply embedded in the atmospheric domain, as discussed in Section 4.4, these methods still fail to provide reliability warnings when physics–data conflicts occur. Conflict detection mechanisms need to be developed for both media. Second, the focus of spatiotemporal interpretation differs. XDL in atmospheric research centers on long-distance transport and regional spatial attribution, with a key focus on resolving the mapping interpretation between satellite column concentration and surface concentration. By comparison, XDL in aquatic research emphasizes the interpretation of temporal heterogeneity driven by hydrological rhythms, clearly distinguishing the switching laws of driving factors between wet and dry seasons. Third, the maturity of multi-task learning varies. Joint modeling of PM_2.5_ and O_3_ has become a standard paradigm in the atmospheric field, with physical constraints resolving inter-task conflicts effectively. In the aquatic field, however, the “negative transfer” issue remains prevalent in multi-task learning; while soft parameter sharing improves model performance, it leads to a sharp surge in the number of parameters.

This divergence points out the direction for cross-medium technology integration: the experience of physical constraint design in the atmospheric field can be transferred to the modeling of advection–diffusion processes in aquatic environments, and the graph neural network-based spatial topology modeling method from aquatic research can optimize the attribution accuracy of irregular monitoring stations in the atmospheric field, ultimately supporting the integrated governance of cross-medium pollution.

## 8. Future Development Directions and Outlook

### 8.1. From Correlation to Causation: Causal Interpretable Deep Learning

Current XDL applications rely heavily on SHAP-based attribution, which remains correlational rather than causal. Policymakers need answers to “whether reducing emission X will inevitably reduce pollutant Y”—a question SHAP cannot answer. A promising direction is integrating causal discovery with deep learning. Fu et al. [88] proposed the CADEPT framework, combining SHAP with LiNGAM to identify causal pathways of air pollution, revealing that temperature and precipitation causally improve air quality, while SO_2_ emissions cause deterioration. For multi-pollutant systems with feedback loops (e.g., O_3_ and PM_2.5_), dynamic Bayesian networks or neural causal models with time-lagged effects are needed [89]. Furthermore, as noted in Section 4.4, PINNs face an “interpretability paradox” when physical residuals conflict with data. One solution is probabilistic causal priors. For example, the advection–diffusion equation is encoded as a probabilistic constraint to reduce the prediction bias of NO_x_ [61]. Future work should combine causal discovery with uncertainty-aware physical constraints to move from “what correlates” to “what causes”. For regulatory applications, causal models should generate actionable statements. For example, Ma et al. [90] employed regression discontinuity causal analysis combined with meteorological normalization to quantify the causal effects and 95% confidence intervals of the sharp road traffic decline on NO_2_, O_3_, and PM_2.5_ concentrations across London during COVID-19 lockdowns. Using SHAP-based interpretable machine learning, they further revealed the spatial contributions to NO_2_ emission reductions, generating actionable causal conclusions and attribution evidence to inform air quality management decisions.

### 8.2. Uncertainty-Aware and Trustworthy Interpretation

Existing XDL methods treat interpretability and uncertainty quantification separately. SHAP values are point estimates, yet model predictions carry substantial uncertainty that varies across pollutants and conditions. As shown in Table 1 and Table 2, spatial cross-validation R^2^ often drops significantly, indicating that interpretations without uncertainty provide false confidence. An integrated framework is needed. Ding et al. [91] constructed a feature-level ensemble framework by integrating multi-scale physical temporal features with XGBoost, and combined it with the bootstrap iteration to generate the PM_2.5_ concentration estimation results for the contiguous United States from 2000 to 2023 with a 95% confidence interval, which were applied to the precise prediction for air quality management and exposure assessment. Conformal prediction offers distribution-free prediction intervals and can be coupled with SHAP [92]. Moreover, SHAP values themselves have uncertainty. Ensemble-based attribution, reporting both central estimates and uncertainty bounds, should become standard. Finally, as highlighted in Section 4.4, when physical constraints and data diverge, models should output a “data–physics consistency index” to warn decision-makers. In terms of specific implementation, an independent module can be added at the model output end to calculate the relative entropy or gradient consistency between the physical residuals and the data fitting residuals, and output an early warning threshold [93]. In practice, regulators can use this consistency index as a red-light/green-light signal: when confidence is low, automated decisions should be suspended, and expert review triggered.

### 8.3. Data Quality, Generative Augmentation, and Standardization

Data scarcity and quality issues pervade multi-pollutant XDL: high missing rates, spatial sparsity, and temporal heterogeneity. Recent advances in generative AI offer solutions. The DGMI framework uses diffusion-based GANs for multivariate air quality imputation, outperforming existing methods by 3–4% in RMSE/MAE, while its discriminator partially addresses the “spurious pattern” problem of SMOTE [94,95]. However, physical consistency (e.g., mass conservation) must be embedded directly into generation. On the standardization front, ISO/IEC TR 20226:2025 provides a framework for AI environmental sustainability, signaling growing regulatory attention. For multi-pollutant XDL, domain-specific standards are needed for: (a) interpretability requirements in environmental decision-making, (b) validation of interpretations against physical mechanisms, and (c) uncertainty reporting alongside SHAP values. Federated learning offers a path to train models across distributed monitoring networks without centralizing raw data, but its interpretability (e.g., decomposing global SHAP into site-specific contributions) remains an open research frontier. To enable cross-jurisdictional regulatory acceptance, future standards must require that data augmentation methods pass physical sanity checks—for example, no generation of “high pesticide with zero rainfall” samples—otherwise interpretations may be legally challengeable.

## 9. Conclusions

XDL advances multi-pollutant synergistic control by enabling nonlinear attribution and physics-informed learning. Yet a deeper, often overlooked problem remains: interpretability lacks a verifiable ground truth. SHAP provides statistical correlations; physics constraints enforce mass conservation or advection–diffusion laws. When they disagree—e.g., monitoring data show a concentration surge but physical equations predict a decline—current XDL has no mechanism to detect, quantify, or signal the conflict. Consequently, users receive “explanations” that may be physically implausible without any warning. This silent inconsistency fundamentally undermines trust in model outputs for policy decisions.

We argue that the next breakthrough is not higher predictive accuracy but verifiable interpretability. Three specific shifts are required:

(1) From correlation-only to causal-aware attribution, achieved by integrating uncertainty-quantified physical priors into interpretable frameworks.
(2) From blind physics–data fusion to conflict-detecting systems that output a “data–physics consistency index” alongside predictions and attributions.
(3) From no evaluation standards to domain-specific validation benchmarks that penalize physically implausible explanations.

For environmental regulators, this means receiving not just SHAP values, but also confidence intervals, consistency scores, and conflict alerts. Such a transition would transform XDL from a post hoc explainer into a dependable decision-support tool for cross-media pollution governance.

## Figures and Tables

**Table 1:** Typical paradigms and key findings of XDL applications in atmospheric multi-pollutant synergistic governance.

Application Paradigm	Core Model Combination	Scientific Question	Key Common Findings/Methodological Advances (Replacing Scattered Accuracy Metrics)	Major Limitations	References
Driving factor attribution	XGBoost/RF + SHAP	Distinguish contributions of meteorology vs. emissions to PM_2.5_/O_3_	Meteorological contribution ~58–70%, but dominant drivers show seasonal shifts; during COVID-19, emission reduction is the main cause of PM_2.5_ decrease, with meteorological change <5%	SHAP values heavily depend on feature independence; collinearity (e.g., temperature vs. seasonal encoding) can lead to false causal inference	[62,63,64]
Synergistic inversion & estimation	SOPiNet/Convtrans/IPMDNN	Real-time high-resolution synchronous inversion of PM_2.5_ and O_3_	Multi-task joint modeling improves R^2^ by 6–9% over single-task models; gain is more significant when missing rate of one pollutant >20%	Uneven generalization; spatial cross-validation R^2^ drops from 0.92 to 0.85, due to overfitting to specific meteorology–emission combinations	[6,65,66]
Toxic component source tracking	XGBoost-SHAP	Identify key components driving oxidative potential of PM_2.5_	Water-soluble organic carbon and K^+^ contribute 25.6% and 22.3%; traditional PMF underestimates due to linear assumptions	Statistical association, not causality; cannot distinguish local emissions from regional transport	[30,67]
Physics-informed fusion	ADRNet/PINN	Embed diffusion equations or mass conservation into predictions	Adding physical residual regularization improves R^2^ by 11–22% in out-of-site tests; “hard constraints” ensure atom conservation but limit generalization	When physics-data conflict occurs, model cannot output a “current explanation is unreliable” confidence	[49,68]

**Table 2:** Typical paradigms and key findings of XDL applications in water multi-pollutant synergistic governance.

Application Paradigm	Core Model Combination	Scientific Question	Key Common Findings/Methodological Advances (Replacing Scattered Accuracy Metrics)	Major Limitations	References
Pollution attribution & source apportionment	XGBoost/RF + SHAP + land use/socioeconomic data	Quantify differentiated contributions of meteorology, land-use, and socioeconomic factors; identify key drivers	Including land-use and socioeconomic variables increases TN/TP prediction R^2^ from 0.17–0.39 to 0.58–0.60 (improvement ~230%); SHAP identifies cropland, forest, and air temperature as top factors for DO, TN, and TP	SHAP provides only statistical association, not causality (e.g., direction between TN and TP); spatial collinearity (GDP vs. built-up area) biases contribution estimates	[14,73,74,75,76,77]
Multi-pollutant/multi-task joint prediction	LSTM-Transformer/CNN-LSTM/multi-output ANN/soft-parameter-sharing networks	Predict multiple water quality parameters simultaneously (TN, TP, DO, COD, etc.) using shared information	Joint modeling improves classification accuracy from 88.7% to 94.6%; CEEMDAN-LSTM-Transformer (decomposing high/low frequencies) achieves R^2^ = 0.87 (+5% over non-decomposed LSTM); soft parameter sharing yields R^2^ = 0.89–0.96 for five parameters	Hard parameter sharing causes negative transfer when task relativity is low (TN NSE drops from 0.84 to 0.68); soft sharing improves performance but dramatically increases parameter count, and lacks physical constraints	[77,78,79,80]
Small-sample & spatial heterogeneity modeling	Graph neural networks (aGNN)/Bayesian neural networks (BNN)/SMOTE augmentation	Pollutant concentration inference under sparse monitoring sites and uncertainty quantification	aGNN achieves R^2^ = 99% on 51 irregular sites, infers unmonitored sites within 500 m of known stations (error < 0.3 mg/L); BNN outputs 95% confidence intervals covering observed extreme concentrations; SMOTE improves AUC for high-concentration samples by 13%	Graph construction (Euclidean distance vs. hydrological topology) is subjective and not quantified; BNN does not incorporate input feature uncertainty (instrument error); SMOTE may generate physically impossible “pseudo-patterns”	[76,81,82,83]
Mechanism–data fusion	SWAT + XGBoost/LSTM + SHAP	Use process-model outputs as inputs to deep learning to enhance interpretability	Using SWAT-simulated lateral flow nitrate load as XGBoost input reduces TP prediction RMSE by 81.9%; SHAP attribution gains process-based support	Remains at the “feature engineering” level, not true physical constraints (e.g., embedding mass conservation in loss function); uncertainty of SWAT parameter calibration is ignored	[84,85]

## Data Availability

No new data were created or analyzed in this study. Data sharing is not applicable to this article.

## References

[1] Goutam Mukherjee A., Ramesh Wanjari U., Eladl M.A., El-Sherbiny M., Elsherbini D.M.A., Sukumar A., Kannampuzha S., Ravichandran M., Renu K., Vellingiri B. (2022). Mixed Contaminants: Occurrence, Interactions, Toxicity, Detection, and Remediation. Molecules.

[2] He C., Liu J., Zhou Y., Zhou J., Zhang L., Wang Y., Liu L., Peng S. (2024). Synergistic PM_2.5_ and O_3_ Control to Address the Emerging Global PM_2.5_-O_3_ Compound Pollution Challenges. Eco-Environ. Health.

[3] Luo B., Chen K., Zheng X., Wu J., Li P., Chen H. (2026). Reactive Transport and Retention of Cd, Pb, and Zn under Coexisting Multimetal-Xanthate Conditions in Porous Media. Water Res..

[4] Zhao Y., Song J., Cheng K., Liu Z., Yang F. (2024). Migration and Remediation of Typical Contaminants in Soil and Groundwater: A State of Art Review. Land Degrad. Dev..

[5] Zheng J., Wang Q., Liu C., Wang J., Liu H., Li J. (2023). Relation Patterns Extraction from High-Dimensional Climate Data with Complicated Multi-Variables Using Deep Neural Networks. Appl. Intell..

[6] Yan X., Zuo C., Li Z., Chen H.W., Jiang Y., He B., Liu H., Chen J., Shi W. (2023). Cooperative Simultaneous Inversion of Satellite-Based Real-Time PM_2.5_ and Ozone Levels Using an Improved Deep Learning Model with Attention Mechanism. Environ. Pollut..

[7] Antamis T., Drosou A., Vafeiadis T., Nizamis A., Ioannidis D., Tzovaras D. (2024). Interpretability of Deep Neural Networks: A Review of Methods, Classification and Hardware. Neurocomputing.

[8] Yang Y., Mei G., Izzo S. (2022). Revealing Influence of Meteorological Conditions on Air Quality Prediction Using Explainable Deep Learning. IEEE Access.

[9] Cremades A., Hoyas S., Deshpande R., Quintero P., Lellep M., Lee W.J., Monty J.P., Hutchins N., Linkmann M., Marusic I. (2024). Identifying Regions of Importance in Wall-Bounded Turbulence through Explainable Deep Learning. Nat. Commun..

[10] Fukushima K. (1980). Neocognitron: A Self-Organizing Neural Network Model for a Mechanism of Pattern Recognition Unaffected by Shift in Position. Biol. Cybern..

[11] Confalonieri R., Coba L., Wagner B., Besold T.R. (2021). A Historical Perspective of Explainable Artificial Intelligence. WIREs Data Min. Knowl. Discov..

[12] Park Y., Kwon B., Heo J., Hu X., Liu Y., Moon T. (2020). Estimating PM_2.5_ Concentration of the Conterminous United States via Interpretable Convolutional Neural Networks. Environ. Pollut..

[13] Mirzavand Borujeni S., Arras L., Srinivasan V., Samek W. (2023). Explainable Sequence-to-Sequence GRU Neural Network for Pollution Forecasting. Sci. Rep..

[14] Zheng H., Liu Y., Wan W., Zhao J., Xie G. (2023). Large-Scale Prediction of Stream Water Quality Using an Interpretable Deep Learning Approach. J. Environ. Manag..

[15] Yan H., Fu H., Chen Z., Liao A.-R., Shen M.-Y., Tao Y., Wu Y.-H., Hu H.-Y. (2025). A Multi-Task Deep Neural Network Reveals Inflowing River Impacts for Predictive Lake Management. Environ. Sci. Ecotechnol..

[16] Ye S., Zeng G., Wu H., Zhang C., Liang J., Dai J., Liu Z., Xiong W., Wan J., Xu P. (2017). Co-Occurrence and Interactions of Pollutants, and Their Impacts on Soil Remediation—A Review. Crit. Rev. Environ. Sci. Technol..

[17] Yan Z., Jin X., Feng C., Leung K.M.Y., Zhang X., Lin Q., Wu F. (2025). Beyond the Single-Contaminant Paradigm: Advancing Mixture Toxicity and Cumulative Risk Assessment in Environmental Toxicology. Environ. Sci. Technol..

[18] Wang Y., Wang Y., Zhang W., Yao X., Wang B., Wang Z. (2022). Spatiotemporal Changes of Eutrophication and Heavy Metal Pollution in the Inflow River System of Baiyangdian after the Establishment of Xiongan New Area. PeerJ.

[19] Lei Y., Wu K., Zhang X., Kang P., Du Y., Yang F., Fan J., Hou J. (2023). Role of Meteorology-Driven Regional Transport on O_3_ Pollution over the Chengdu Plain, Southwestern China. Atmos. Res..

[20] Ivatt P.D., Evans M.J., Lewis A.C. (2022). Suppression of Surface Ozone by an Aerosol-Inhibited Photochemical Ozone Regime. Nat. Geosci..

[21] Hargreaves A.J., Vale P., Whelan J., Alibardi L., Constantino C., Dotro G., Cartmell E., Campo P. (2018). Impacts of Coagulation-Flocculation Treatment on the Size Distribution and Bioavailability of Trace Metals (Cu, Pb, Ni, Zn) in Municipal Wastewater. Water Res..

[22] Kuhn L., Beirle S., Kumar V., Osipov S., Pozzer A., Bösch T., Kumar R., Wagner T. (2024). On the Influence of Vertical Mixing, Boundary Layer Schemes, and Temporal Emission Profiles on Tropospheric NO_2_ in WRF-Chem—Comparisons to in Situ, Satellite, and MAX-DOAS Observations. Atmos. Chem. Phys..

[23] Zhang C., Fu T. (2023). Recalibration of a Three-Dimensional Water Quality Model with a Newly Developed Autocalibration Toolkit (EFDC-ACT v1.0.0): How Much Improvement Will Be Achieved with a Wider Hydrological Variability?. Geosci. Model Dev..

[24] Yi M., Lin F. (2025). A Hybrid Air Quality Prediction Method Based on VAR and Random Forest. J. Comput. Commun..

[25] Liu N., Hao Z., Zhao P. (2024). Spatiotemporal Change of PM_2.5_ Concentration in Beijing-Tianjin-Hebei and Its Prediction Based on Machine Learning. Urban Clim..

[26] Hou Q., Sun Z., He L., Karemat A. (2022). Orthogonal Grid Physics-Informed Neural Networks a Neural Network-Based Simulation Tool for Advection–Diffusion–Reaction Problems. Phys. Fluids.

[27] Seo B., Li J. (2024). Explainable Machine Learning by SEE-Net: Closing the Gap between Interpretable Models and DNNs. Sci. Rep..

[28] Lemhadri I., Ruan F., Abraham L., Tibshirani R. (2021). LassoNet: A Neural Network with Feature Sparsity. J. Mach. Learn. Res..

[29] Kim G., Heo G. (2023). Solving Partial Differential Equation for Atmospheric Dispersion of Radioactive Material Using Physics-Informed Neural Network. Nucl. Eng. Technol..

[30] Esu C.O., Pyo J., Cho K. (2024). Machine Learning-Derived Dose-Response Relationships Considering Interactions in Mixtures: Applications to the Oxidative Potential of Particulate Matter. J. Hazard. Mater..

[31] Ribeiro M., Singh S., Guestrin C. (2016). “Why Should I Trust You?”: Explaining the Predictions of Any Classifier. Proceedings of the 2016 Conference of the North American Chapter of the Association for Computational Linguistics: Demonstrations, San Diego, CA, USA, 12—17 June 2016.

[32] Nathvani R., Vishwanath D., Clark S.N., Alli A.S., Muller E., Coste H., Bennett J.E., Nimo J., Moses J.B., Baah S. (2023). Beyond Here and Now: Evaluating Pollution Estimation across Space and Time from Street View Images with Deep Learning. Sci. Total Environ..

[33] Liu Y., Yu W., Gao C., Chen M. (2022). An Auto-Extraction Framework for CEP Rules Based on the Two-Layer LSTM Attention Mechanism: A Case Study on City Air Pollution Forecasting. Energies.

[34] Houdou A., El Badisy I., Khomsi K., Abdala S.A., Abdulla F., Najmi H., Obtel M., Belyamani L., Ibrahimi A., Khalis M. (2024). Interpretable Machine Learning Approaches for Forecasting and Predicting Air Pollution: A Systematic Review. Aerosol Air Qual. Res..

[35] Lundberg S., Lee S.-I. (2017). A Unified Approach to Interpreting Model Predictions. arXiv.

[36] Vega García M., Aznarte J.L. (2020). Shapley Additive Explanations for NO2 Forecasting. Ecol. Inform..

[37] Tao C., Zhang Q., Huo S., Ren Y., Han S., Wang Q., Wang W. (2024). PM_2.5_ Pollution Modulates the Response of Ozone Formation to VOC Emitted from Various Sources: Insights from Machine Learning. Sci. Total Environ..

[38] Wang C., Zhang S., Zhang Y., Cui S., Qin X., Guenther A., Bai J., Gu D., Du J., Tang J. (2026). VOCs-Driven Ozone Extremes during Dry and Wet Heatwaves in the Jiangsu–Shandong–Henan–Anhui Boundary: Integrating Meteorological Forcings and SHAP Interpretation. Atmos. Res..

[39] Chen H., Covert I.C., Lundberg S.M., Lee S.-I. (2023). Algorithms to Estimate Shapley Value Feature Attributions. Nat. Mach. Intell..

[40] Chen H., Lundberg S.M., Lee S.-I. (2022). Explaining a Series of Models by Propagating Shapley Values. Nat. Commun..

[41] Wikle C.K., Datta A., Hari B.V., Boone E.L., Sahoo I., Kavila I., Castruccio S., Simmons S.J., Burr W.S., Chang W. (2023). An Illustration of Model Agnostic Explainability Methods Applied to Environmental Data. Environmetrics.

[42] Tasioulis T., Bagkis E., Kassandros T., Karatzas K. (2025). The Quest for the Best Explanation: Comparing Models and XAI Methods in Air Quality Modeling Tasks. Appl. Sci..

[43] Longo L. (2023). Explainable Artificial Intelligence: First World Conference, xAI 2023, Lisbon, Portugal, July 26–28, 2023, Proceedings, Part I.

[44] Zhang H., Huan J., Xu X., Shi B., Zheng Y., Mao J., Lv J. (2023). Model Evaluation of Total Phosphorus Prediction Based on Model Accuracy and Interpretability for the Surface Water in the River Network of the Jiangnan Plain, China. Water Sci. Technol..

[45] Herbinger J., Wright M.N., Nagler T., Bischl B., Casalicchio G. (2024). Decomposing Global Feature Effects Based on Feature Interactions. J. Mach. Learn. Res..

[46] Rajakumari R Peter R., Ambadas Lanjewar U. (2023). Improving Air Quality Prediction with a Hybrid Bi-LSTM and GAN Model. ECTI Trans. Comput. Inf. Technol..

[47] Abdulameer Y.H., Ibrahim A.A. (2025). A Hybrid Model Using 1D-CNN with Bi-LSTM, GRU, and Various ML Regressors for Forecasting the Conception of Electrical Energy. Int. J. Mod. Phys. C.

[48] Daw A., Yeo K., Karpatne A., Klein L. (2022). Multi-Task Learning for Source Attribution and Field Reconstruction for Methane Monitoring. 2022 IEEE International Conference on Big Data (Big Data).

[49] Zakariaei N., Rout S., Haber E., Eliasof M. Advection Augmented Convolutional Neural Networks. Proceedings of the 38th Annual Conference on Neural Information Processing Systems (NeurIPS 2024).

[50] Chen Y., Xu Y., Wang L., Li T. (2023). Modeling Water Flow in Unsaturated Soils through Physics-Informed Neural Network with Principled Loss Function. Comput. Geotech..

[51] Zhao C., Lin Z., Yang L., Jiang M., Qiu Z., Wang S., Gu Y., Ye W., Pan Y., Zhang Y. (2025). A Study on the Impact of Meteorological and Emission Factors on PM_2.5_ Concentrations Based on Machine Learning. J. Environ. Manag..

[52] Kanayankottupoyil J., Mohammed A.A., John K. (2025). Hybrid Deep Learning Framework for Forecasting Ground-Level Ozone in a North Texas Urban Region. Appl. Sci..

[53] Keerthana A.V., Pragna K.P., Suhani S., Surabhi A.S., Sattigeri S.K., Raju N.V. (2024). Predicting PM_2.5_ Concentrations in Bengaluru Using Ensemble Machine Learning Models and Explainable AI Techniques. 2024 8th International Conference on Computational System and Information Technology for Sustainable Solutions (CSITSS).

[54] Chen T.-F., Gong X.-L., Tsai C.-Y., Chang K.-H. (2025). Method for Planning Subarea Emission Reduction Strategies to Improve Ozone over a Large Area: A Case of Taiwan. Atmos. Environ..

[55] Hong-Zhao D., Hong-Mei G., Shi-Kai L., Fang Y., Qiang Y. (2025). Synergistic Effect Evaluation Method of Atmospheric Emission Reduction Based on Deep Learning Fusion Model. J. Hazard. Mater..

[56] Khodakarami S., Oommen V., Daryakenari N.A., Beekenkamp M., Karniadakis G.E. (2026). Spectral Bias in Physics-Informed and Operator Learning: Analysis and Mitigation Guidelines. arXiv.

[57] Wang S., Teng Y., Perdikaris P. (2020). Understanding and Mitigating Gradient Pathologies in Physics-Informed Neural Networks. arXiv.

[58] Naser M.Z. (2026). Fundamental Flaws of Physics-Informed Neural Networks and Explainability Methods in Engineering Systems. Comput. Ind. Eng..

[59] Gothwal R., Thatikonda S. (2020). Modeling Transport of Antibiotic Resistant Bacteria in Aquatic Environment Using Stochastic Differential Equations. Sci. Rep..

[60] Zhang Z., Ma X., Schlesinger D. (2026). Interpretable Air Pollution Forecasting by Physics-Guided Spatiotemporal Decoupling. arXiv.

[61] Li L., Khalili R., Lurmann F., Pavlovic N., Wu J., Xu Y., Liu Y., O’Sharkey K., Ritz B., Oman L. (2025). Knowledge-Informed Deep Learning to Mitigate Bias in Joint Air Pollutant Prediction. Environ. Int..

[62] Zhang L., Wang L., Ji D., Xia Z., Nan P., Zhang J., Li K., Qi B., Du R., Sun Y. (2024). Explainable Ensemble Machine Learning Revealing the Effect of Meteorology and Sources on Ozone Formation in Megacity Hangzhou, China. Sci. Total Environ..

[63] Jiang B., Kong D., Liu H., Cai Y., Shi Y. (2025). Revealing the Drivers of PM_2.5_ Pollution by Explainable Machine Learning Pre- to Post-COVID-19 in “Urumqi-Changji-Shihezi” Region, China. Atmos. Res..

[64] Li T., Zhang Q., Peng Y., Guan X., Li L., Mu J., Wang X., Yin X., Wang Q. (2023). Contributions of Various Driving Factors to Air Pollution Events: Interpretability Analysis from Machine Learning Perspective. Environ. Int..

[65] Ren Y., Wang S., Xia B., Xia B. (2025). Knowledge Based Convolutional Transformer for Joint Estimation of PM_2.5_ and O_3_ Concentrations. Sci. Rep..

[66] Chen B., Hu J., Wang Y., Feng T., Sun W., Feng Z., Yang G., Wang H. (2025). An Interpretable Physics-Informed Deep Learning Model for Estimating Multiple Air Pollutants. GISci. Remote Sens..

[67] Li R., Yan C., Meng Q., Yue Y., Jiang W., Yang L., Zhu Y., Xue L., Gao S., Liu W. (2024). Key Toxic Components and Sources Affecting Oxidative Potential of Atmospheric Particulate Matter Using Interpretable Machine Learning: Insights from Fog Episodes. J. Hazard. Mater..

[68] Li L., Wang J., Franklin M., Yin Q., Wu J., Camps-Valls G., Zhu Z., Wang C., Ge Y., Reichstein M. (2023). Improving Air Quality Assessment Using Physics-Inspired Deep Graph Learning. npj Clim. Atmos. Sci..

[69] Shi H., Yang N., Yang X., Tang H. (2023). Clarifying Relationship between PM_2.5_ Concentrations and Spatiotemporal Predictors Using Multi-Way Partial Dependence Plots. Remote Sens..

[70] Sturm P.O., Wexler A.S. (2022). Conservation Laws in a Neural Network Architecture: Enforcing the Atom Balance of a Julia-Based Photochemical Model (v0.2.0). Geosci. Model Dev..

[71] Amato F., Van Drooge B.L., Jaffrezo J.L., Favez O., Colombi C., Cuccia E., Reche C., Ippolito F., Ridolfo S., Lara R. (2024). Aerosol Source Apportionment Uncertainty Linked to the Choice of Input Chemical Components. Environ. Int..

[72] De Foy B., Edwards R., Joy K.S., Zaman S.U., Salam A., Schauer J.J. (2024). Interpretable Machine Learning Tools to Analyze PM_2.5_ Sensor Network Data so as to Quantify Local Source Impacts and Long-Range Transport. Atmos. Res..

[73] Yuan Y., Zhou C., Wu J., Deng F., Liu W., Sun M., Li L. (2025). An Interpretable Deep Learning Framework for River Water Quality Prediction—A Case Study of the Poyang Lake Basin. Water.

[74] Zhao Y.-L., Sun H.-J., Wang X.-D., Ding J., Lu M.-Y., Pang J.-W., Zhou D.-P., Liang M., Ren N.-Q., Yang S.-S. (2024). Spatiotemporal Drivers of Urban Water Pollution: Assessment of 102 Cities across the Yangtze River Basin. Environ. Sci. Ecotechnol..

[75] Wang F., Wang Y., Zhang K., Hu M., Weng Q., Zhang H. (2021). Spatial Heterogeneity Modeling of Water Quality Based on Random Forest Regression and Model Interpretation. Environ. Res..

[76] Ban M.J., Lee D.H., Lee B.-T., Kang J.-H. (2024). Assessing the Environmental Determinants of Micropollutant Contamination in Streams Using Explainable Machine Learning and Network Analysis. Chemosphere.

[77] Yao J., Chen S., Ruan X. (2024). Interpretable CEEMDAN-FE-LSTM-Transformer Hybrid Model for Predicting Total Phosphorus Concentrations in Surface Water. J. Hydrol..

[78] Helaly M.A., Rady S., Mabrouk M., Aref M.M., Villarroya S., Cotos J.M. (2025). HAXNet: A Hybrid Dual Attention Cross-Stitch Network for Multi-Task Water Quality Forecasting. Proceedings of the 2025 7th Novel Intelligent and Leading Emerging Sciences Conference (NILES), Giza, Egypt, 25–27 October 2025.

[79] Kothari E., Mary S., Pandit J., Kamesh D., K. P., Abbas S. (2025). Real-Time and Explainable Hybrid Machine Learning Framework for Multivariate Prediction and Classification of Water Contamination Using Environmental and Temporal Features. Proceedings of the 1st International Conference on Research and Development in Information, Communication, and Computing Technologies.

[80] Gorgoglione A., Russo C., Gioia A., Iacobellis V., Castro A. (2025). Comparing Neural Network Architectures for Simulating Pollutant Loads and First Flush Events in Urban Watersheds: Balancing Specialization and Generalization. Chemosphere.

[81] Kang X., Kokkinaki A., Shi X., Lee J., Guo Z., Ni L., Wu J. (2024). Modeling Upscaled Mass Discharge From Complex DNAPL Source Zones Using a Bayesian Neural Network: Prediction Accuracy, Uncertainty Quantification and Source Zone Feature Importance. Water Resour. Res..

[82] Pang M., Du E., Zheng C. (2024). Contaminant Transport Modeling and Source Attribution With Attention-Based Graph Neural Network. Water Resour. Res..

[83] Guo Y., Cui Y., Chen H., Xie J., Zhang C., Liu J. (2024). Self-Potential Inversion Based on Attention U-Net Deep Learning Network. J. Cent. South Univ..

[84] Jeong D.S., Jeong H., Kim J.H., Kim J.H., Park Y. (2024). A Hybrid Approach to Improvement of Watershed Water Quality Modeling by Coupling Process–Based and Deep Learning Models. Water Environ. Res..

[85] Huan J., Fan Y., Xu X., Zhou L., Zhang H., Zhang C., Hu Q., Cai W., Ju H., Gu S. (2025). Deep Learning Model Based on Coupled SWAT and Interpretable Methods for Water Quality Prediction under the Influence of Non-Point Source Pollution. Comput. Electron. Agric..

[86] Wan H., Xiang L., Cai Y., Xie Y., Xu R. (2025). Temporal and Spatial Feature Extraction Using Graph Neural Networks for Multi-Point Water Quality Prediction in River Network Areas. Water Res..

[87] Elreedy D., Atiya A.F., Kamalov F. (2024). A Theoretical Distribution Analysis of Synthetic Minority Oversampling Technique (SMOTE) for Imbalanced Learning. Mach. Learn..

[88] Fu Z., Yang X., Ma Y., Sun Y., Wang T. (2025). Integrating Explainable AI and Causal Inference to Unveil Regional Air Quality Drivers in China. J. Environ. Manag..

[89] He C., Ren J., Liu W. (2023). Dynamic Causal Modeling and Online Collaborative Forecasting of Air Quality in Hong Kong and Macao. Entropy.

[90] Ma L., Graham D.J., Stettler M.E.J. (2023). Using Explainable Machine Learning to Interpret the Effects of Policies on Air Pollution: COVID-19 Lockdown in London. Environ. Sci. Technol..

[91] Ding Y., Dong J., Teng M., Meng S., Yang J., Li S. (2026). A Feature-Level Ensemble Framework for Improving Daily PM_2.5_ Estimation across the Contiguous United States (2000–2023). Environ. Model. Softw..

[92] Hajibabaee P., Behnood A., Ngo T., Mohammadi Golafshani E. (2025). Carbonation Depth Assessment of Recycled Aggregate Concrete: An Application of Conformal Prediction Intervals. Expert Syst. Appl..

[93] Barajas-Solano D.A. (2026). Statistical Learning Analysis of Physics-Informed Neural Networks. arXiv.

[94] Cheng N., Ni Q. (2025). DGMI: A Diffusion-Based Generative Adversarial Framework for Multivariate Air Quality Imputation. Appl. Intell..

[95] Alahyari S., Domaratzki M. (2025). SMOGAN: Synthetic Minority Oversampling with GAN Refinement for Imbalanced Regression. arXiv.

